# Contour integration and aging: the effects of element spacing, orientation alignment and stimulus duration

**DOI:** 10.3389/fpsyg.2013.00356

**Published:** 2013-06-20

**Authors:** Eugenie Roudaia, Patrick J. Bennett, Allison B. Sekuler

**Affiliations:** ^1^Vision and Cognitive Neuroscience Lab, Department of Psychology, Neuroscience and Behaviour, McMaster UniversityHamilton, ON, Canada; ^2^Institute of Neuroscience, Multisensory Cognition Research Group, Trinity College DublinDublin, Ireland

**Keywords:** aging, contour integration, orientation, collinearity, perceptual grouping, distracters, suppression

## Abstract

The ability to extract contours in cluttered visual scenes, which is a crucial step in visual processing, declines with healthy aging, but the reasons for this decline are not well understood. In three experiments, we examined how the effect of aging on contour discrimination varies as a function of contour and distracter inter-element spacing, collinearity, and stimulus duration. Spiral-shaped contours composed of Gabors were embedded within a field of distracter Gabors of uniform density. In a four alternative forced-choice task, younger and older subjects were required to report the global orientation of the contour. In Experiment 1, the absolute contour element spacing varied from two to eight times the Gabor wavelength and contour element collinearity was disrupted with five levels of orientation jitter. Contour discrimination accuracy was lower in older subjects, but the effect of aging did not vary with contour spacing or orientation jitter. Experiment 2 found that decreasing stimulus durations from 0.8 to 0.04 s had a greater effect on older subjects' performance, but only for less salient contours. Experiment 3 examined the effect of the background on contour discrimination by varying the spacing and orientation of the distracter elements for contours with small and large absolute spacing. As in Experiment, the effect of aging did not vary with absolute contour spacing. Decreasing the distracter spacing, however, had a greater detrimental effect on accuracy in older subjects compared to younger subjects. Finally, both groups showed equally high accuracy when all distracters were iso-oriented. In sum, these findings suggest that aging does not affect the sensitivity of contour integration to proximity or collinearity. However, contour integration in older adults is slower and is especially vulnerable when distracters are denser than contour elements.

## Introduction

Extracting contours is an important step in the process of translating incoming visual information into a meaningful percept. Because visual scenes often contain multiple objects that overlap and partially occlude each other, grouping different features into contours is not a trivial task, as features belonging to a single contour need to be grouped with one another and not grouped with other objects, or with elements of the background. Contours in natural scenes contain important statistical regularities (Geisler et al., 2001; Sigman et al., 2001; Elder and Goldberg, 2002) and human observers appear to use the statistical properties of contours in natural images to detect contours (Field et al., 1993; Hess et al., 2003; Geisler and Perry, 2009).

The rules governing contour grouping have been studied successfully using variations on the path paradigm (Field et al., 1993), in which subjects detect a contour whose path is defined by a group of discrete, oriented elements (e.g., Gabors) that are embedded within a field of similar distracters. Several studies have shown that contour saliency strongly depends on the alignment of local element orientations along the contour path and, to a lesser extent, on the separation of contour elements (Field et al., 1993; Kovács and Julesz, 1993; Saarinen and Levi, 2001). Other factors known to influence contour detection are contour curvature, length, eccentricity, spatial scale and phase alignment of contour elements (Hess and Dakin, 1997; Dakin and Hess, 1998; Beaudot and Mullen, 2001, 2003; Hess et al., 2003; Ledgeway et al., 2005; Kuai and Yu, 2006), as well as characteristics of the surrounding context, closure, and contour object identity (Kovács and Julesz, 1993; Braun, 1999; Mathes and Fahle, 2007; Dakin and Baruch, 2009; Nygård et al., 2011).

Neuroimaging studies in humans and primates have revealed contour-specific activity both in the primary visual cortex and extrastriate visual areas (Altmann et al., 2003; Kourtzi et al., 2003; Kourtzi and Huberle, 2005; Tanskanen et al., 2008). Neurophysiological studies using single-cell recordings have demonstrated response facilitation of V1 neurons to oriented lines presented in the context of a contour (Kapadia et al., 1995; Li et al., 2006). More recently, Gilad et al. (2013) used voltage-sensitive dye imaging to examine the population responses of primary visual neurons of monkeys engaged in a contour detection task. Their findings revealed that, whereas the initial rise in activity in response to the stimulus is not affected by the presence of a contour, the later part of the neural response follows the perceptually-grouped percept: activation to the contour region increased, while activation throughout the background region was suppressed. Moreover, this difference in activity across the stimulated region was correlated with behavioral performance in a contour detection task, providing strong evidence that activity in primary visual cortex underlies perceptual grouping of contours. Finally, the contour-related modulation of V1 responses has been shown to be affected by learning, spatial attention, and task demands (Gilbert et al., 2000; Li et al., 2008; Gilad et al., 2013), indicating that top-down feedback from higher-order areas plays an important role in perceptual grouping (Angelucci and Bullier, 2003; Ciaramelli et al., 2007; Verghese, 2009; Volberg et al., 2013).

Healthy aging is accompanied by changes in many aspects of visual function, but the changes are not uniform: some functions remain completely unimpaired, while others decline rapidly (for reviews see Spear, 1993; Sekuler and Sekuler, 2000; Faubert, 2002). The intriguing variation in the effects of aging in vision may be due to differences in the level of processing complexity required in different stimuli and tasks (Habak and Faubert, 2000; Faubert, 2002), and the availability of compensatory mechanisms to mask the sensory and perceptual deficits (McIntosh et al., 1999; Della-Maggiore et al., 2000; Bennett et al., 2001). Thus, age-related changes in visual function may be more pronounced in situations where multiple stages of processing contribute to successful performance, as is the case in contour integration.

Growing evidence suggests that contour integration declines with healthy aging (Del Viva and Agostini, 2007; Roudaia et al., 2008; McKendrick et al., 2010; Roudaia et al., 2011; Casco et al., 2011). Del Viva and Agostini (2007) first showed that older subjects were able to tolerate fewer distracters than younger subjects when detecting closed circular contours embedded among distracters. Roudaia et al. (2011) found that older subjects required longer stimulus durations than younger subjects to successfully discriminate the location of the gap in a “C”-shaped contour embedded among distracters. McKendrick et al. (2010) used closed circular contours to examine the effect of aging on shape discrimination; Contrary to what was found in previous studies, McKendrick et al. found that the ability to discriminate closed contour shapes was only slightly (and not significantly) worse in older subjects. Moreover, the addition of background elements or the addition of orientation jitter to contour elements did not have a differential effect on the two age groups. However, McKendrick et al. did find that older subjects required more contour elements (or smaller contour spacing) than younger subjects to make the contour shape discrimination, suggesting that older subjects had greater difficulty extracting the shape of contours containing large inter-element spacings. Casco et al. (2011) measured thresholds in a task that required subjects to detect the radial displacement of a single Gabor belonging to a circular contour. Consistent with McKendrick et al. (2010), thresholds for older and younger subjects were not different when the contour was composed of aligned Gabors and presented without distracters. However, older subjects required a larger displacement than younger subjects when the contour was composed of Gabors alternating between aligned and orthogonal orientations, or when the contour was embedded among randomly-oriented distracter Gabors.

The reasons for the observed age-related changes in contour integration are not well understood. Previous research has demonstrated that performance in contour integration tasks requires the integration of contour elements, as well as the suppression of the distracters in the background (Dakin and Baruch, 2009; Sassi et al., 2010; Machilsen et al., 2011; Schumacher et al., 2011). Casco et al. (2011) argued that the ability to integrate contour elements together did not change with aging, and that the age-related deficit in contour detection and discrimination resulted primarily from a decreased ability to suppress distracters with irrelevant orientations. Although this hypothesis is consistent with the pattern of results obtained in their task, and with evidence of reductions in inhibitory mechanisms in the visual cortex (e.g., Leventhal et al., 2003; Fu et al., 2010), there is also evidence that contour grouping abilities decline with aging even in the absence of visual clutter (Roudaia et al., 2008), a finding that is difficult to explain by deficits in suppressive mechanisms alone.

In the following studies, we further characterized the effects of aging on contour integration by examining contour discrimination performance for a range of stimuli varying in contour and distracter inter-element spacing and local orientation alignment. Contour element spacing is known to affect contour detection (Kovács and Julesz, 1993; Li and Gilbert, 2002; Beaudot and Mullen, 2003; Watt et al., 2008) and it has been suggested that contour integration mechanisms operate over a limited spatial range that scales with the spatial frequency of the contour elements (Beaudot and Mullen, 2003). In Experiment 1, we examined whether the spatial range of contour integration changes with aging by measuring contour discrimination performance for contours with varying inter-element spacing. Moreover, we examined the sensitivity to contour element collinearity as a function of contour spacing by disrupting the alignment of contour element orientations with orientation jitter. Knowing that older subjects require longer stimulus durations than younger subjects to integrate contours (Roudaia et al., 2011), any effects of aging on performance in Experiment 1 were expected to depend on stimulus duration. To examine this relationship, a subset of representative conditions from Experiment 1 were repeated in Experiment 2 with a range of stimulus durations. In addition to contour element spacing, the relative spacing of contour and distracter elements is also known to affect contour detection (Kovács et al., 1999; Li and Gilbert, 2002). Therefore in Experiment 3 we examined the effect of relative contour and distracter spacing on contour discrimination in older and younger subjects. Considering previous evidence that older subjects experience greater impairments in performance in the presence of visual noise or clutter (e.g., Sekuler and Ball, 1986; Betts et al., 2005; Del Viva and Agostini, 2007; Rousselet et al., 2009), changes in relative spacing may have a greater effect on older subjects. Finally, we also measured contour discrimination against a background of iso-oriented Gabors to examine whether aging also affects the ability to segregate contours from a relatively uniform background.

In all three experiments, the stimuli consisted of spiral shaped contours sampled with Gabor elements and embedded in a uniform field of distracter Gabors. Subjects were required to report the global orientation of the spiral contour on every trial in a four alternative forced-choice task. Spiral contours were chosen because (1) we wanted to avoid closed or circular contours, due to possible additional detection benefits for closure and circularity (Regan and Hamstra, 1992; Kovács and Julesz, 1993; Dumoulin and Hess, 2007); (2) we wanted to use smooth contours that would be comparable to previous studies that have used open “C”-shaped contours (Roudaia et al., 2008, 2011) and closed circular and ellipsoid shapes (Del Viva and Agostini, 2007; Casco et al., 2011; Hadad, 2012); (3) using a familiar spiral shape allowed us to devise an easily-understood task that assessed global contour discrimination, as opposed to contour detection; and (4) drawing spiral contours, as opposed to straight contours, allowed for a greater range of contour spacings.

## Experiment 1: effects of contour element spacing and local orientation alignment

This experiment examined the effects of contour element separation and local orientation alignment on contour integration in younger and older subjects. The separation between adjacent elements comprising the contour was varied across blocks, while the relative contour and distracter separation was kept constant. Within blocks, alignment of contour element orientations was manipulated by the addition of varying amounts of orientation jitter.

### Methods

#### Subjects

Seventeen younger (*M* = 25 years; range: 22–33) and 16 older (*M* = 66 years; range: 60–82) subjects participated in this study and were compensated at a rate of $10/h. Near and far visual acuities were measured in all subjects using the SLOAN Two Sided ETDRS Near Point Test and the 4 Meter 2000 Series Revised ETDRS charts (Precision Vision, LaSalle, Illinois, USA). Contrast sensitivity was estimated using the Pelli-Robson Contrast Sensitivity Test (Pelli et al., 1988). Subjects wore their habitual optical correction during the vision testing and during the experiment. All subjects had normal or corrected-to-normal near and far Snellen visual acuity (range: −0.29 to 0.16 logMAR), although, on average, older subjects showed poorer acuity than younger subjects. All subjects showed normal contrast sensitivity for their age group (Elliott et al., 1990; Mäntyjärvi and Laitinen, 2001). The mini-mental state examination assessment (Folstein et al., 1975) was used to screen for cognitive impairment among older subjects and all scored above the normal cut-off score of 25/30. All subjects were free of visual pathology such as cataracts, glaucoma, and retinopathy, as assessed by a self-report questionnaire. One subject underwent successful bilateral cataract surgery 4 years prior to the experiment. Table 1 summarizes the relevant demographic information for the two groups.

**Table 1:** **Mean ± 1 SD age, near and far logMAR acuity, Pelli-Robson contrast sensitivity, and mini-mental state examination (MMSE)**.

**N (M:F)**	**Age**	**Near acuity**	**Far acuity**	**Pelli-Robson**	**MMSE**
17 (11:6)	24.9 ± 3.20	−0.15 ± 0.08	−0.11 ± 0.09	1.95 ± 0.05	
16 (9:7)	65.9 ± 6.37	0.03 ± 0.11	−0.05 ± 0.08	1.92 ± 0.06	28.9 ± 1.06

#### Apparatus

The experiment was programmed using the Psychophysics and Video Toolboxes (v. 3; Brainard, 1997; Pelli, 1997) in the Matlab environment (v. 7.5) driven by a Macintosh G5 computer. The stimuli were presented on a 20-inch (51 cm) Sony Trinitron monitor with a 1280 × 1024 resolution (pixel size: 0.014°) and a refresh rate of 75 Hz. The display was the only light source in the room and had an average luminance of 52 cd/m^2^. Subjects viewed the display binocularly through natural pupils from a viewing distance of 114 cm. Viewing position was stabilized with a chin/forehead rest.

#### Stimuli

**Figure 1:**
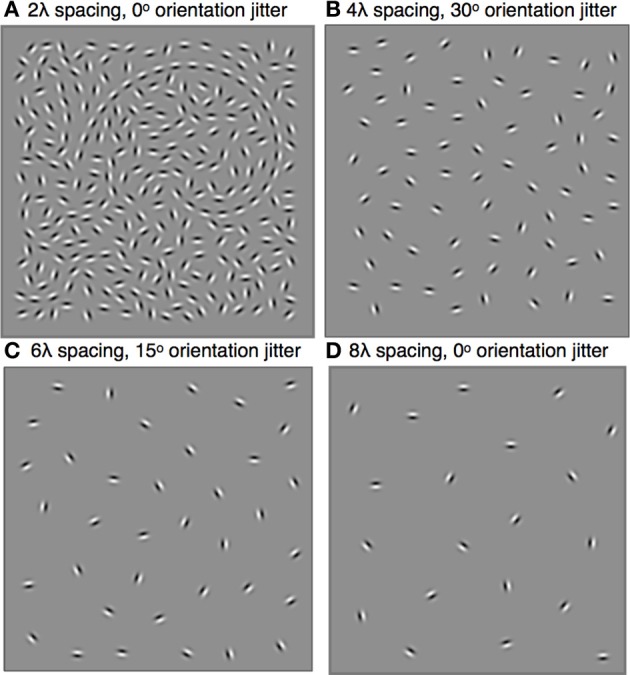
**Stimuli were spiral-shaped contours sampled with Gabors (λ = 0.30°, σ = 0.11°) and embedded in a field of randomly oriented distracter Gabors**. The shape and size of the spiral, which could be clockwise or counter-clockwise, remained constant throughout the experiment, but its global orientation and position varied randomly across trials. Subjects reported the global orientation of the spiral by indicating the location of its tail. The minimum inter-element distance varied across blocks and ranged from 0.6° to 2.4°, or 2λ to 8λ. Within each block, contour element orientations were jittered by the addition of local orientation jitter ranging from 0° to 60°. Four stimulus examples are shown: **(A)** 2λ distance, 0° jitter, tail is on the left; **(B)** 4λ distance, 30° jitter, tail is on the right; **(C)** 6λ spacing, 15° jitter, tail is at the top; **(D)** 8λ spacing, 0° jitter, tail is at the bottom.

The stimulus consisted of a spiral-shaped contour sampled with Gabor micropatterns and embedded in a square region (9.5 × 9.5°) of randomly oriented distracter Gabors (Figure 1). Gabor micropatterns were created by multiplying a 3.33 cycles/deg sine wave grating (λ = 0.30°, 20 pixels/cycle) of 90% contrast by a circular Gaussian envelope with a standard deviation (σ) of 0.11° (≈1.4 visible cycles). All Gabors were in positive sine phase with respect to the center of the Gaussian window. The shape of the contour was defined by the formula for a logarithmic spiral,

(1) $$r=ae^{b\text{(}t+t_{j}\text{)}}$$

where *a* = 1.18°, *b* = 0.20, and 1.25 < *t* < 3. The position of the first Gabor on the contour relative to the beginning of the spiral path was jittered by parameter *t*_*j*_, a uniform random variable ranging from −0.1 to 0.1. Subsequent Gabors were placed at the appropriate locations along the spiral path such that the inter-element distance between all elements remained constant. The long axis of the spiral spanned ≈6.9°.

The center of the spiral was placed in the center of the 9.5° square and then displaced horizontally and vertically by random amounts selected from a Normal distribution with σ = 1.5°. An iterative procedure was used to fill the remaining portion of the square region with distracter Gabors. Gabors were placed in random locations subject to the contstraint that all neighboring Gabors were not closer than a pre-determined minimum inter-element distance and the procedure continued until no more elements could be placed (Dakin and Baruch, 2009). On half the trials, the pattern was reflected along the vertical axis to create clockwise and counterclockwise spirals. Finally, the pattern was rotated by 90°, 180°, or 270° to create spirals of four different global orientations (see Figure 1).

The inter-element distance between neighboring Gabor elements was varied across blocks. Inter-element distance were set to be 2, 4, 6, or 8 times the Gabor wavelength (λ), corresponding to 0.6, 1.2, 1.8, or 2.4°. Setting the minimum distance between distracter elements equal to the contour inter-element spacing created, by definition, a background with the smallest number of distracters needed to avoid any density or spacing cues to the location of the contour. The approximate ratios of the number of contour and distractor Gabors for the 2λ, 4λ, 6λ, and 8λ inter-element spacings were 26:240, 13:60, 9:25, and 7:14 Gabors, respectively. Due to the random nature of the iterative process, the number of background elements varied slightly from trial to trial.

For each inter-element spacing condition, contour discrimination performance was measured for different levels of contour element collinearity. Collinearity was manipulated by adding varying amounts of orientation jitter to the contour element orientations. Collinear (or aligned) contours were created by setting the orientation of each element to equal the tangent to the contour path at that position. An independent orientation jitter angle was then added to each element by picking random numbers from a uniform distribution spanning ranges of ±15°, ±30°, ±45°, or ±60°.

#### Procedure

The McMaster University Research Ethics Board approved the experimental protocol. Written informed consent was obtained from all subjects prior to their participation in the experiment.

A four-alternative forced-choice (4-AFC) procedure was used to measure contour discrimination accuracy. A stimulus containing a spiral contour was presented on every trial and subjects were asked to report whether the tail of the spiral was located in the top-center, bottom-center, right-middle, left-middle locations in the display. At the beginning of the experiment, each subject was shown examples of stimuli where the spiral contour was made clearly visible by reducing the contrast of background elements. After adapting to the luminance of the display for 60 s, subjects completed five practice trials. The experimenter ensured that all subjects understood the task before proceeding to the experimental trials.

Each trial began with a black fixation point (diameter = 0.12°) presented in the center of the blank screen of mean luminance. Subjects were instructed to fixate the fixation point, which remained in the center throughout the trial. The fixation point flickered a rate of 10 Hz for 0.3 s at the beginning of each trial, after which the stimulus array was presented for 1 s, followed by a blank screen for 0.5 s. The fixation point was then displayed until the subject's response. The global orientation of the spiral was randomized across trials and the subjects reported the location of the end of the spiral by pressing either the up, down, left, or right arrow key. Auditory feedback was given on every trial with a high pitch tone indicating a correct response and a low pitch tone indicating an error. The subsequent trial began after a 1.5 s inter-trial interval. The four inter-element distance conditions were tested in separate blocks in randomized order. Each block consisted of 25 trials at orientation jitter levels of 0°, ±15°, ±30°, ±45°, and ±60°.

#### Preliminary control experiment

The stimuli and task used in the main experiments assessed subjects' ability to perceive the spiral's global shape/orientation by requiring them to report the location of the spiral's tail. To examine whether this task could be performed on the basis of local contour information, a preliminary control experiment measured the minimum number of contour elements required to locate the tail of the spiral contour.

The stimuli and methods used in that control experiment were created using the same procedure described above, except that the distracter elements were removed and, on every trial, a staircase procedure determined the number of adjacent contour elements that would be displayed. The location of the adjacent elements on the spiral path was chosen randomly on every trial to be either the head, tail, or middle of the spiral. The two young subjects who participated in this experiment were shown the full shape of the spiral prior to the experiment and were told that only portions of the spiral contour would be visible on any given trial. Their task was to report whether the tail of the complete spiral would be at the top-center, bottom-center, right-middle, and left-middle locations of the square pattern.

### Results

For all of the current experiments, statistical analyses were performed using the statistical computing environment R (R Development Core Team, 2008; Lawrence, 2011). Mean accuracy and sensitivity (i.e., *d*′) were obtained for each level of orientation jitter and inter-element distance. Since the pattern of results was similar for accuracy and *d*′, only accuracy data are presented here. When conducting ANOVA, an arcsine transformation was applied to accuracy values to ensure the data satisfied the assumption of normality. For all within-subjects tests, the Geisser-Greenhouse correction was used to adjust the degrees-of-freedom to correct for violations of the sphericity assumption (Maxwell and Delaney, 2004). In such cases, the adjusted *p*-values are reported. Generalized eta-squared, η^2^_*g*_, is reported as a measure of association strength for all significant effects (Olejnik and Algina, 2003; Bakeman, 2005).

Our preliminary experiment found that 11 Gabors were required to locate the tail for contours with 2λ spacing, and 4.5 Gabors were required for contours with 6λ spacing. Thus, reporting the location of the tail of the spiral (without distracters) with 75% accuracy in our stimuli requires grouping approximately half of the contour elements composing the contour.

**Figure 2:**
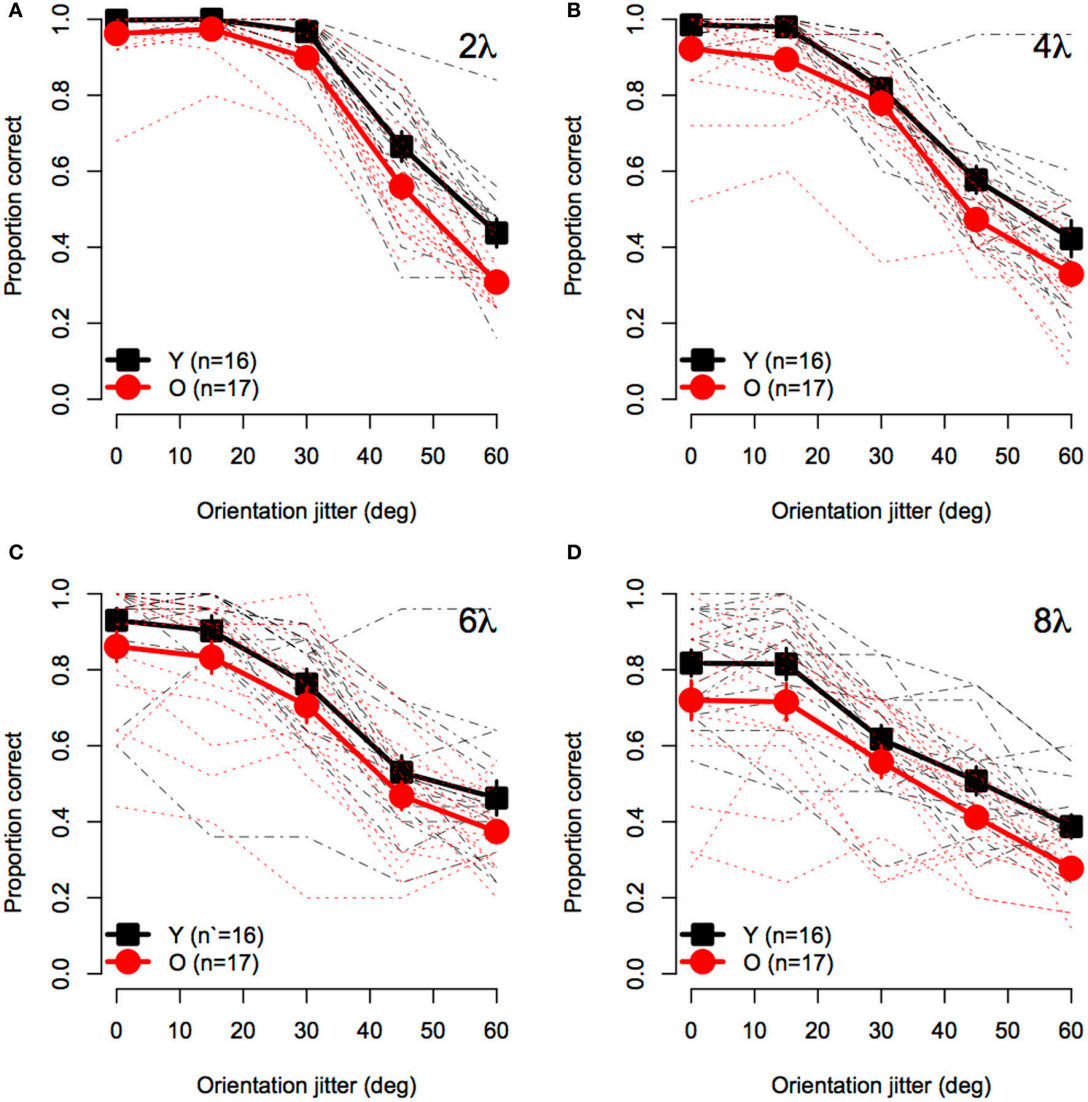
**Experiment 1 results: Contour discrimination accuracy is shown as a function of contour element orientation jitter for four inter-element spacing conditions (A–D)**. Black squares show mean accuracy of younger subjects and red circles show mean accuracy of older subjects. Error bars represent ±1 SEM. Individual subjects' data are shown in black dashed line for younger subjects and dotted red lines for older subjects.

Figure 2 shows mean accuracy for contour discrimination as a function of orientation jitter for all contour spacing conditions separately. As expected, accuracy in both groups declined with increasing orientation jitter at each level of element spacing. Furthermore, at any fixed level of orientation jitter, response accuracy in both groups decreased with increasing separation between elements. Accuracy in both groups was at ceiling when discriminating aligned (0° jitter) contours with 2λ spacing. However, accuracy was lower in older subjects than younger subjects in all other conditions. A mixed-model 2 (Age) × 4 (Spacing) × 5 (Orientation Jitter) ANOVA revealed significant main effects of age [*F*_(1, 31)_ = 10.5, *p* = 0.0028, η^2^_*g*_ = 0.10], spacing [*F*_(3, 93)_ = 54.4, $\hat{\epsilon }$ = 0.92, *p* < 0.0001, η^2^_*g*_ = 0.29], and orientation jitter [*F*_(4, 124)_ = 480.3, $\hat{\epsilon }$ = 0.49, *p* < 0.0001, η^2^_*g*_ = 0.72]. The Spacing × Orientation Jitter interaction also was significant [*F*_(12, 372)_ = 17.0, $\hat{\epsilon }$ = 0.63, *p* < 0.0001, η^2^_*g*_ = 0.14], indicating that the effect of orientation jitter varied with inter-element spacing. Importantly, none of the interactions with age were significant [Age × Spacing: *F*_(3, 93)_ = 0.08, $\hat{\epsilon }$ = 0.92, *p* = 0.96; Age × Orientation Jitter: *F*_(4, 124)_ = 0.53, $\hat{\epsilon }$ = 0.49, *p* = 0.57; Age × Spacing × Orientation Jitter: *F*_(12, 372)_ = 0.74, $\hat{\epsilon }$ = 0.63, *p* = 0.65]. These results indicate that the effect of age on contour integration did not vary significantly with inter-element spacing or orientation jitter.

**Figure 3:**
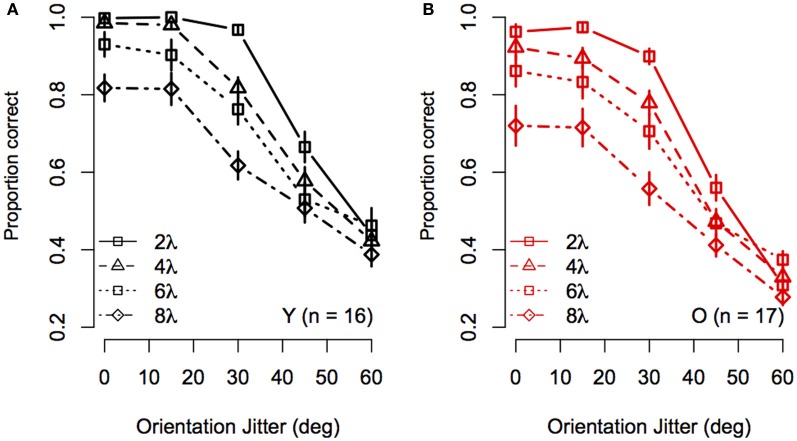
**Experiment 1 results: Effect of inter-element spacing on contour discrimination accuracy is shown as a function of orientation jitter for younger subjects (A) and older subjects (B)**. Different symbols show accuracy for 2λ to 8λ conditions separately (see legend). Error bars represent ±1 SEM.

To visualize the Spacing × Orientation Jitter interaction, the data were redrawn in Figure 3 to compare the effect of orientation jitter on contours with different inter-element spacings. The data show that accuracy achieved with low levels of jitter decreased with increasing inter-element spacing, but that the effect of spacing declined as jitter increased beyond ≈30°. In other words, contour discrimination showed greater resistance to small amounts of orientation jitter when inter-element separations were small.

**Figure 4:**
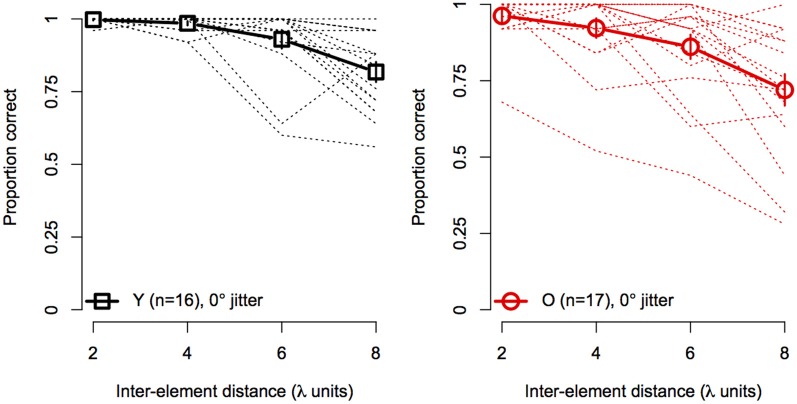
**Experiment 1 results: contour discrimination accuracy is shown a function of inter-element distance for contours with 0° orientation jitter (collinear)**. Dashed lines show individual subjects' data, solid lines show mean accuracy, and error bars represent ±1 SEM.

Figure 4 shows the effect of element spacing for collinear contours (i.e., 0° orientation jitter) for younger and older subjects separately. The average accuracy in the two groups declined monotonically with increasing spacing and the rate of decline was similar for both groups. However, not all subjects showed the same rate of decline with spacing. As can be seen in Figure 4, several subjects in each group showed consistently high accuracy at all spacings. On the other hand, other subjects showed sharp declines in accuracy, even with spacing as small as 4 λ. Moreover, the number of subjects who showed poor performance (≤75%) at larger spacings was greater in the older group than in the younger group.

### Discussion

This experiment examined the effects of contour-element separation and collinearity on contour discrimination in younger and older subjects. Overall, contour discrimination was poorer in older subjects, but the age difference did not vary significantly with element separation or collinearity.

Field et al. (1993) found that contour detection accuracy declined to chance level with orientation jitter of only 30°, whereas subjects in the current experiment were able to tolerate much greater levels of orientation jitter. The reason for this discrepancy lies in the definitions of orientation jitter used in the two studies: A 30° orientation jitter in Field et al. signifies that each contour element's orientation either increased or decreased by 30°, whereas 30° orientation jitter in our experiment means that the jitter angle applied to any individual contour element can take on any value between −30° and 30°. Hence, the effects of orientation jitter in the two studies are not comparable.

Beaudot and Mullen (2003) estimated the spatial limit of contour integration by determining the maximum inter-element distance at which observers could reliably detect contours embedded in a field of randomly oriented Gabors. They found that the critical separation was proportional to spatial frequency and was unaffected by contour curvature. The average spatial limit for the achromatic mechanism of their four younger subjects was ≈6.8λ. Kuai and Yu (2006) reported a similar estimate (6.9λ) in a contour detection task and a smaller (4.4 λ) limit in a contour shape discrimination task. Although the maximum element separation in the current experiment was 8λ, we did not observe a sharp decline in performance at the largest contour spacing in either group. Instead, average contour discrimination performance decreased gradually with element spacing, with some subjects showing a steeper decline and others maintaining high discrimination accuracy even at 8λ. Interestingly, Beaudot and Mullen (2003) also reported that some subjects showed constant performance as a function of contour-element separation. Thus, the spatial range for contour integration may not have a sharp boundary and may vary across individuals. However, if the spatial range of contour integration was reduced with aging, the age-difference in contour discrimination accuracy would be greater for contours with larger spacings, compared to smaller spacings. This pattern was not observed as the effect of age was approximately constant across all contour spacing conditions. Hence, the spatial range of contour integration does not appear change with age.

Consistent with the idea that proximity increases overall contour salience (Li and Gilbert, 2002), the asymptotic level of performance decreased with increasing contour spacing for younger and older subjects. Contours with small spacing were also more resistant to orientation jitter than contours with large spacing.

These results are consistent with previous studies showing that co-alignment of local orientation is required to group elements spaced far apart, but is less important for grouping elements that are placed close together (Nikolaev and van Leeuwen, 2007; Hadad et al., 2010). In the current study, the effect of orientation jitter was the same for younger and older subjects across all contour spacing conditions. This finding is consistent with McKendrick et al. (2010), who found that orientation jitter increased closed-contour shape discrimination thresholds by similar amounts in younger and older subjects. Similarly, Hadad (2012) found that disruption of collinearity had a similar effect on closed contour shape discrimination in younger and older subjects for stimuli with high and low proximity levels. In sum, converging evidence from several studies shows that aging does not affect the ability to use collinearity for grouping contours and extracting the contour shape.

## Experiment 2: effect of stimulus duration as a function of contour spacing and local orientation alignment

Previous research has shown that older subjects require longer stimulus durations than younger subjects to discriminate contours (Roudaia et al., 2011). A 1 s stimulus duration was used in Experiment 1 to ensure that older subjects had sufficient time to group the contours, so that the effects of contour spacing and collinearity could be observed independently of differences in processing time. Processing time for detecting contours in clutter increases with curvature (Beaudot and Mullen, 2001; Hess et al., 2001) and appears to increase with contour element spacing (Beaudot and Mullen, 2003). Similarly, the stimulus duration required for collinear facilitation—i.e., the reduction of contrast threshold for a target Gabor when flanked by high contrast, collinear flanker Gabors (Polat and Sagi, 1993)—has been shown to increase proportionally to the distance between target and flankers (Cass and Spehar, 2005). Thus, the dynamics of contour discrimination in Experiment 1 may also vary with inter-element separation and collinearity, and these dynamics may change with age in a non-linear way. In the current experiment, we tested contour discrimination for a range of stimulus durations to examine how the effect of aging for contours with different spacing and collinearity levels may be affected by the stimulus duration.

### Methods

#### Subjects

Fourteen younger and fourteen older subjects who participated in Experiment 1 were tested. Subjects were compensated ($10/h) for participating. One older subject completed only three out of four conditions in the experiment. For this reason, data from this subject were excluded from statistical analyses, however, they are included in the data plots. Demographic information is presented in Table 1.

#### Apparatus

The apparatus was the same as in Experiment 1.

#### Stimuli

The stimuli were generated using the same procedure as described in Experiment 1. Only two levels of inter-element spacing (2λ and 6λ) and two levels of orientation jitter (0° and 30°) were used in this experiment.

#### Procedure

The task and trial sequence was the same as in Experiment 1, except that the stimulus duration was randomized on every trial. The stimulus durations were 0.04, 0.093, 0.20, 0.40, or 0.80 s (i.e., 3, 7, 15, 30, or 60 frames). The trials were blocked by inter-element spacing and orientation jitter and the order of the blocks was randomized across subjects. Each block contained 150 trials, with 30 trials at each stimulus duration. As in Experiment 1, accuracy and *d*′ were calculated for each subject. The pattern of results was similar for accuracy and *d*′ and only accuracy data are shown here.

### Results

**Figure 5:**
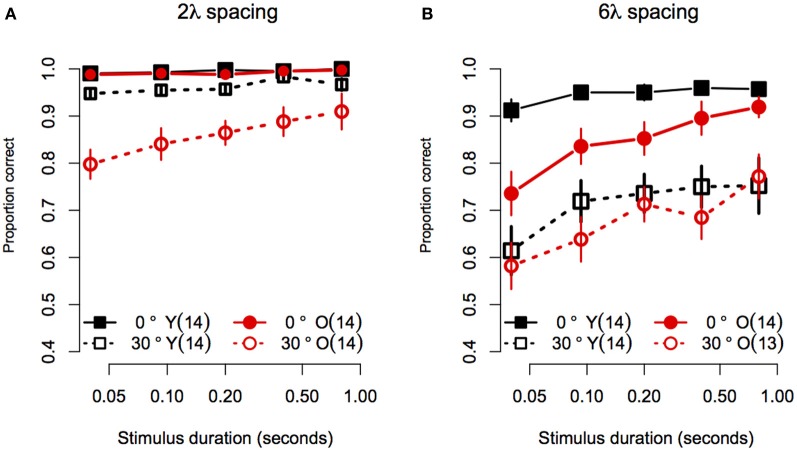
**Experiment 2 results: Contour discrimination accuracy is shown as a function of stimulus duration for (A) contours with small contour element spacing and (B) contour with large contour element spacing**. Younger subjects' mean accuracy is plotted in black squares and older subjects' mean accuracy is plotted in red circles. Error bars represent ±1 SEM.

Statistical analyses were performed with the same software and procedures used in Experiment 1. Figure 5 shows the contour discrimination accuracy of younger and older subjects as a function of stimulus duration for stimuli with small (Figure 5A) and large (Figure 5B) inter-element separations. As can be seen, accuracy generally increased with stimulus duration, but the rate of increase and the asymptotic level of performance varied as a function of age, inter-element separation, and orientation jitter. A mixed-model 2 (Age) × 4 (Spacing) × 5 (Orientation Jitter) × 5 (duration) ANOVA on arcsine-transformed accuracy values revealed significant main effects of age [*F*_(1, 25)_ = 5.78, *p* = 0.02, η^2^_*g*_ = 0.09], spacing [*F*_(1, 25)_ = 219.9, *p* < 0.0001, η^2^_*g*_ = 0.45], orientation jitter [*F*_(1, 25)_ = 232.1, *p* < 0.001, η^2^_*g*_ = 0.43], and duration [*F*_(4, 100)_ = 26.4, $\hat{\epsilon }$ = 0.80, *p* < 0.0001, η^2^_*g*_ = 0.10]. The effects of stimulus duration, contour spacing, and orientation jitter also interacted with each other, as revealed by significant Spacing × Orientation jJitter [*F*_(1, 25)_ = 9.79, *p* = 0.004, η^2^_*g*_ = 0.03], Spacing × Duration [*F*_(4, 100)_ = 4.10, $\hat{\epsilon }$ = 0.76, *p* = 0.009, η^2^_*g*_ = 0.01], and Spacing × Orientation Jitter × Duration [*F*_(4, 100)_ = 3.33, $\hat{\epsilon }$ = 0.84, *p* = 0.02, η^2^_*g*_ = 0.009] interactions. The Orientation Jitter × Duration interaction was not significant [*F*_(4, 100)_ = 1.68, $\hat{\epsilon }$ = 0.87, *p* = 0.16, η^2^_*g*_ = 0.004]. The significant three-way Spacing × Orientation Jitter × Duration interaction reflects the fact that the effect of spacing was greater for non-collinear contours, especially at short stimulus durations.

Consistent with Experiment 1, the effect of age did not vary with spacing or orientation jitter, as the Age × Spacing [*F*_(1, 25)_ = 0.06 *p* = 0.81, η^2^_*g*_ ≈ 0], and Age × Orientation Jitter [*F*_(1, 25)_ = 0.64, *p* = 0.43, η^2^_*g*_ = 0.002] interactions were not significant. Unlike what was found in Experiment 1, the Age × Spacing × Orientation Jitter interaction was significant [*F*_(1, 25)_ = 12.3, *p* = 0.002, η^2^_*g*_ = 0.04]. This result reflects the fact that disruption of collinearity had a greater effect in younger subjects in the 6λ compared to the 2λ condition. On the other hand, adding orientation jitter affected older subjects' accuracy approximately to the same extent in the small and large spacing conditions (compare the difference in the vertical separation of the two black curves in Figures 5A,B versus the same comparison in the red curves). However, it is likely that this interaction is due, at least in part, to ceiling effects in the no-jitter, 2λ conditions.

Importantly, the effect of age also varied with stimulus duration, as revealed by a significant Age × Duration interaction [*F*_(4, 100)_ = 2.97, $\hat{\epsilon }$ = 0.80, *p* = 0.02, η^2^_*g*_ = 0.01]. None of the three- and four-way interactions that included Age and Duration factors were significant [Age × Spacing × Duration: *F*_(4, 100)_ = 0.29, $\hat{\epsilon }$ = 0.76, *p* = 0.88; Age × Orientation Jitter × Duration: *F*_(4, 100)_ = 1.94, $\hat{\epsilon }$ = 0.87, *p* = 0.11; Age × Spacing × Orientation Jitter × Duration: (*F*_(4, 100)_ = 1.90, $\hat{\epsilon }$ = 0.84, *p* = 0.12, η^2^_*g*_ = 0.005)].

Given that the effect of age interacted with spacing and orientation jitter, we conducted separate 2 (Age) × 5 (Duration) ANOVAs for each contour type. In addition, the effect of stimulus duration was analysed by computing linear trend scores of accuracy across log stimulus duration, and comparing these trend scores across age groups with One-Way ANOVAs. Figure 5A shows the data for stimuli with a small (2λ) inter-element spacing. For collinear contours, both groups showed ceiling performance for all durations: average response accuracy in the two groups did not differ [Age: *F*_(1, 25)_ = 1.50, *p* = 0.23, η^2^_*g*_ = 0.03], the linear trend of accuracy across duration did not reach significance [*F*_(1, 25)_ = 4.01, *p* = 0.06], and the linear trend did not differ between age groups [*F*_(1, 25)_ = 0.03, *p* = 0.59]. When collinearity was disrupted by 30° orientation jitter, response accuracy in the two age groups differed at all stimulus durations except the longest (i.e., 0.8 s): average response accuracy was lower in older subjects [*F*_(1, 25)_ = 11.25, *p* = 0.003, η^2^_*g*_ = 0.23], the linear trend of accuracy across duration was significant [*F*_(1, 25)_ = 44.8, *p* < 0.0001]. Importantly, the linear trend differed between age groups [*F*_(1, 25)_ = 5.60, *p* = 0.03], reflecting the fact that response accuracy in older subjects declined more than that of younger subjects as duration decreased. In summary, for contours with short inter-element spacings, the effect of aging on contour discrimination accuracy increased at shorter stimulus durations, but only for non-collinear contours.

Figure 5B shows the data for stimuli with a large (6λ) inter-element spacing. For collinear contours, younger subjects' accuracy improved from 91 to 95% between 0.04 and 0.093 s, after which it remained constant as stimulus duration increased. On the other hand, older subjects' accuracy was ≈73% when the stimulus duration was 0.04 s, and showed an approximately linear increase until reaching younger subjects' accuracy at 0.8 s. Consistent with these observations, the main effect of age was significant [*F*_(1, 25)_ = 7.11, *p* = 0.01, η^2^_*g*_ = 0.16], the linear trend across log duration was significant [*F*_(1, 25)_ = 39.35, *p* < 0.0001] and the linear trend was greater in older compared to younger subjects [*F*_(1, 25)_ = 7.72, *p* = 0.01]. For non-collinear (30° jitter) contours, both groups showed similar levels of accuracy at all durations. The main effect of age was not significant [*F*_(1, 25)_ = 0.48, *p* = 0.49, η^2^_*g*_ = 0.01]. The linear trend across log duration was significant [*F*_(1, 25)_ = 20.60, *p* = 0.0001], but it did not differ across the two age groups [*F*_(1, 25)_ = 0.32, *p* = 0.57]. In summary, for contours with 6λ spacing, the effect of aging increased with decreasing stimulus duration when contour elements were collinear, but the effect of aging remained constant for non-collinear contours.

### Discussion

When spiral contours were composed of closely spaced collinear elements, younger and older subjects showed nearly perfect contour discrimination performance, even when stimulus duration was decreased to 0.04 s. However, contour discrimination accuracy declined with decreasing stimulus duration when contour element spacing was large and when contour element collinearity was disrupted by local orientation jitter. When both proximity and collinearity were disrupted (6λ and 30° jitter condition), both groups showed similar, poor performance at all durations. However, when only proximity *or* collinearity was disrupted, performance was more severely impaired in older subjects than younger subjects at short stimulus durations.

These findings are consistent with a previous study showing an age-related increase in duration thresholds for discriminating contours in clutter (Roudaia et al., 2011). Roudaia et al. demonstrated that this age difference in processing time could not be ascribed to age-related reductions in retinal illuminance (Weale, 1963), nor to delays in the processing of individual Gabor elements, and therefore argued that the age difference in contour discrimination reflected delays in the contour integration process *per se*. Similar to the current results, Roudaia et al. found that older subjects were disproportionately slower at processing less salient contours composed of elements oriented orthogonally to the contour path.

Roudaia et al., (2011) reported that older subjects required longer durations to process collinear contours than younger subjects, and that younger and older subjects in that study required 0.05 and 0.150 s, respectively to discriminate contours with 75% accuracy. It may be surprising, therefore, that the current study found that response accuracy in both age groups was quite high at short stimulus durations. However, the difference between experiments is likely due to the fact that stimuli used by Roudaia et al. were followed by a mask, whereas those used in the current study were not. This hypothesis is supported by Hess et al., (2001), who found that successful contour detection required a stimulus duration ≥ 0.83 s when stimuli were preceded and followed by a mask, but only 0.013 s when stimuli were not masked.

The current data suggest that processing time for contour integration increases with increasing inter-element spacing. Subjects in both groups showed a steeper decrease in accuracy with decreasing stimulus duration for contours with large, compared to small, inter-element spacings. The only other study that measured contour detection as a function of stimulus duration and inter-element separation was conducted in macaque monkeys and also reported increased processing time for integrating across larger separations (Mandon and Kreiter, 2005).

Finally, consistent with Experiment 1, the effect of aging on discrimination of contours did not systematically differ as a function of contour spacing in this experiment. This result corroborates the conclusion made in Experiment 1, namely that aging does not differentially affect the ability to group contours across large distances.

## Experiment 3: the effect of relative contour and distracter spacing in ISO- and randomly-oriented backgrounds

In Experiments 1 and 2, the average spacing between adjacent distracters and between adjacent contour elements were equal in order to minimize the number of distracters while simultaneously ensuring that the contour could not be detected on the basis of density cues. Previous studies have shown that contour detection becomes more difficult when the minimum distracter element spacing is less than the minimum contour element spacing, resulting in displays where any given contour element is closer to a distracter than to their neighboring contour element (Braun, 1999; Kovács et al., 1999; Li and Gilbert, 2002). Del Viva and Agostini (2007) found that younger subjects are able to tolerate a greater number of distracter Gabors than older subjects when detecting circular contours, which suggests that older subjects are less efficient than younger subjects at extracting contours from dense backgrounds. However, the stimuli in that study did not equate the average spacing between elements across the display, resulting in differences in local density that may have been used to locate the contour. In this experiment, we investigated the effect of relative spacing of contour and distracter elements on contour discrimination in younger and older subjects by decreasing distracter spacing while keeping the spacing between contour elements constant. In addition, we examined whether contour integration in older subjects is differentially affected by the variability of the orientations of the background elements.

### Methods

#### Subjects

Twelve younger and twelve older subjects, none of whom had participated in Experiments 1 or 2, were recruited to participate in this experiment. Subjects were compensated for their time at a rate of $10/h. Table 2 summarizes the demographic factors, visual acuity, contrast sensitivity, and cognitive measures for these two groups.

**Table 2:** **Experiment 3: Mean ± 1 SD age, near and far logMAR acuity, Pelli-Robson contrast sensitivity, and mini-mental state examination (MMSE)**.

**N (M:F)**	**Age**	**Near acuity**	**Far acuity**	**Pelli-Robson**	**MMSE**
12 (4:8)	20.0 ± 3.5	−0.12 ± 0.07	−0.12 ± 0.11	1.90 ± 0.07	
12 (6:6)	67.6 ± 6.5	0.03 ± 0.08	0.01 ± 0.09	1.95 ± 0	28.8 ± 1.03

#### Apparatus

The apparatus was the same as in Experiment 1.

#### Stimuli

Spiral contours were sampled with Gabors (λ = 0.30°, σ = 0.11°) and positioned at equally spaced intervals of 3λ (0.9°) or 6λ (1.8°) along the path of a logarithmic spiral, as described in Experiment 1. The global spiral orientation was either clockwise or counterclockwise and was oriented in one of four directions. The spiral was centered in a 9.5° square region and then displaced horizontally and vertically by random amounts selected from a Normal distribution with σ = 1.5°. The orientations of the Gabors composing the spiral were tangential to the contour path.

**Figure 6:**
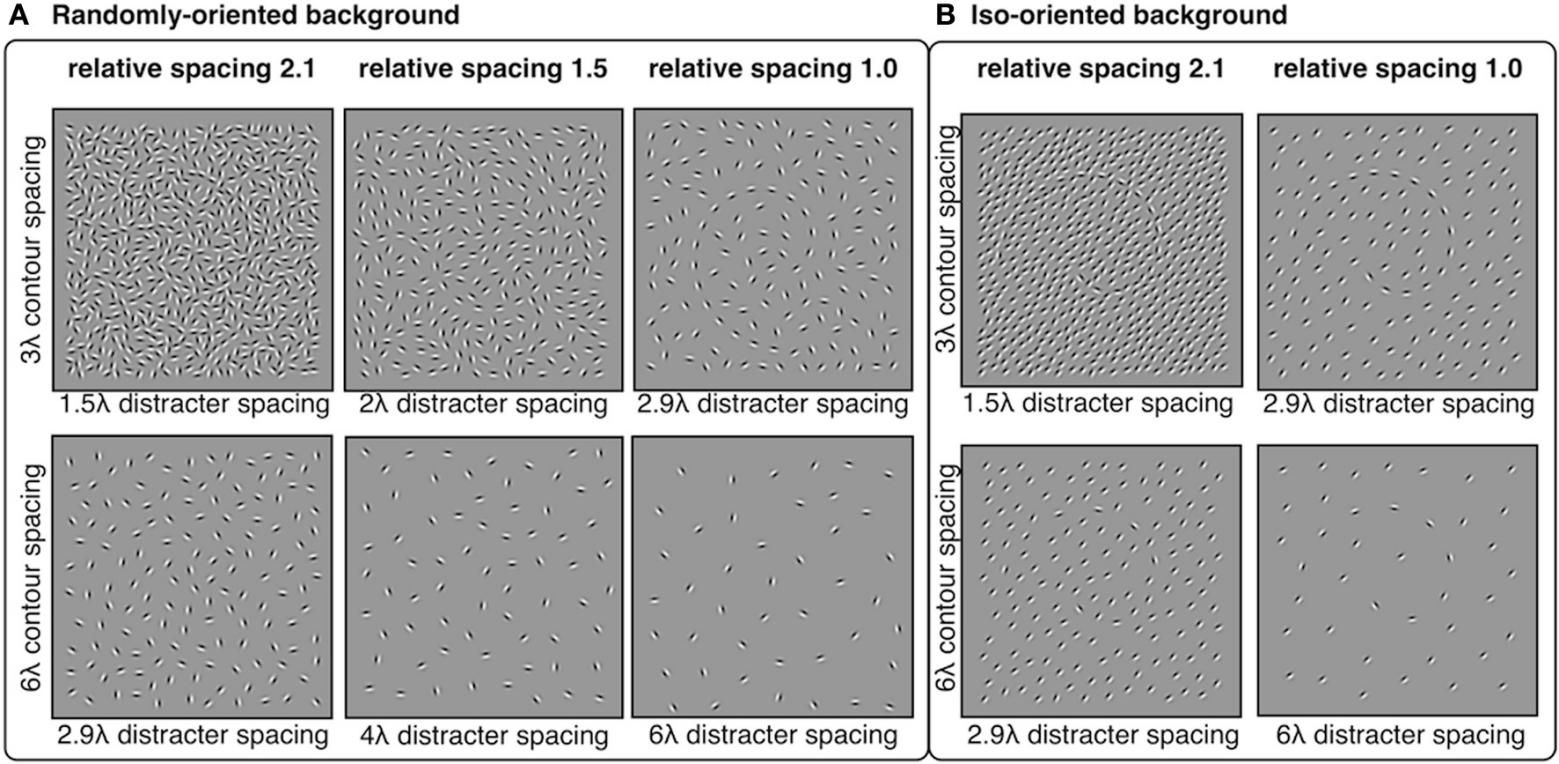
**Experiment 3 stimuli: spiral contours embedded in a background of randomly-oriented (A) or iso-oriented (B) Gabors with varying inter-element spacing**. Contour elements had either 3λ (top row) or 6λ (bottom row) spacing. Distracter spacing varied from 1.5λ to 6.1λ, resulting in contour-distracter relative spacing ranging from 1.0 to 2.1. Three levels of relative spacing—1, 1.5, and 2.1—are shown here.

Distracter elements were positioned randomly within the square stimulus area using an iterative procedure that maintained a pre-determined minimum separation between distracter elements. The iterative procedure continued until no more distracters could be placed. The 3λ contours were embedded in backgrounds with minimum distracter spacing of 2.9, 2.4, 2.0, 1.7, and 1.5λ. The 6λ contours were embedded in backgrounds with minimum distracter spacing of 6.1, 5.0, 4.0, 3.4, and 2.9λ. Thus, the relative spacing between contour and distracter elements were equal to 1.0, 1.2, 1.5, 1.8, or 2.1, where numbers greater than 1 indicate that contour spacing was larger than distracter spacing. The orientation of distracter elements varied in two conditions: random and iso-oriented. In the random condition, distacter orientations were sampled from a uniform distribution of angles from 0° to 360°. In the iso-oriented condition, each distracter orientation was sampled from a Gaussian normal distribution with σ = 5° and a mean that was equal to a randomly chosen angle between 0° and 360°. The iso-oriented background was tested only for relative spacing levels of 1.0 and 2.1, and the randomly-oriented background was tested with all five levels of relative spacing. Examples of stimuli are shown in Figure 6.

#### Procedure

The task and trial sequence were similar to Experiments 1 and 2. Subjects were asked to maintain fixation on a black fixation point displayed in the middle of the screen of mean luminance throughout the trials. The fixation point flickered to indicate the start of each trial. The stimulus contained a spiral contour oriented in one of four directions, was displayed for 0.4 s and followed by a blank screen of mean luminance for 0.5 s. In a four alternative forced-choice procedure, subjects reported the location of the tail of the spiral by pressing one of the four arrow keys on the keyboard. Auditory feedback was provided after every trial with a high pitch tone following a correct response and a low pitch tone following an error. The subsequent trial began after a 1.5 s inter-trial interval.

Trials were blocked by contour spacing: half the subjects in each group completed all the 3λ contour trials first and the other half of the subjects completed all the 6λ contour trials first. Within each block, all relative spacing levels and background context conditions (iso- or randomly-oriented) were intermixed. There were 50 trials per condition, resulting in a total of 600 trials. Subjects were allowed to take a short break after every 125 trials and the experiment lasted approximately 50 min.

### Results

**Figure 7:**
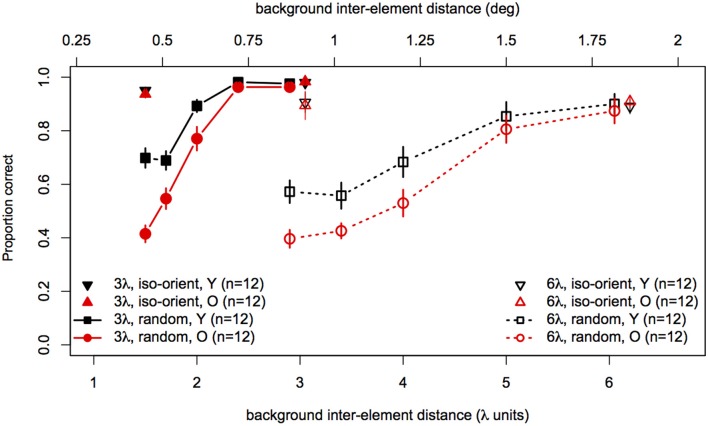
**Experiment 3 results: contour discrimination accuracy is shown as a function of background inter-element spacing for all conditions tested**. Data for the iso-oriented background conditions are shown by the triangle symbols (up, black for younger subjects and down, red for older subjects). Black squares and red circles show younger and older subjects' performance in the random background condition. Solid lines and filled symbols show data for contours with 3λ spacing and dotted lines with open symbols show data for contours with 6λ spacing. Error bars represent ±1 SEM.

**Figure 8:**
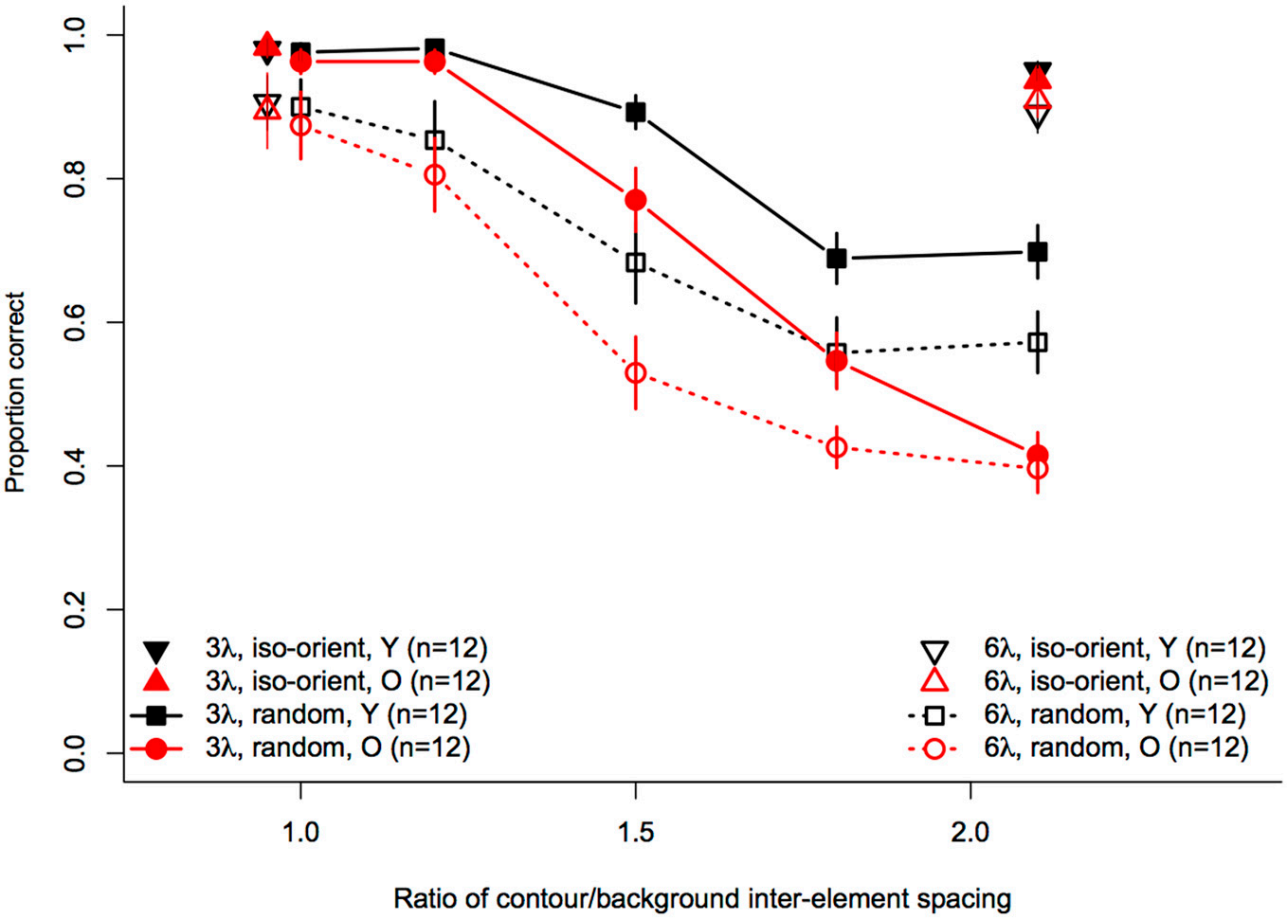
**Experiment 3 results: contour discrimination accuracy is shown as a function of relative contour and background spacing**. Data for the iso-oriented background conditions are shown by the triangle symbols (up, black for younger subjects and down, red for older subjects) that are displaced horizontally to avoid overlap. Data for the random orientation condition background condition are shown with filled symbols and solid lines for the 3λ contours and with open symbols and dashed lines for the 6λ contours. Error bars represent ±1 SEM.

Statistical analyses were performed with the same software and procedures used in Experiments 1 and 2. Figure 7 shows contour discrimination accuracy as a function of background element spacing and Figure 8 shows the same data as a function of the relative spacing of contour and background elements.

To compare performance in the iso-oriented (upright and inverted triangles) and random background conditions (circles and squares), we conducted a 2 (Age) × 2 (Context: random or iso-oriented) × 2 (Contour Spacing: 3λ and 6λ) × 2 (Relative Spacing: 1.1 and 2.2) mixed-model ANOVA on arcsine-transformed accuracy values. The ANOVA revealed significant main effects of background context [*F*_(1, 22)_ = 230, *p* < 0.001], contour spacing [*F*_(1, 22)_ = 19.3, *p* = 0.0002], and relative spacing [*F*_(1, 22)_ = 201.6, *p* < 0.0001], but no main effect of age [*F*_(1, 22)_ = 2.3, *p* = 0.14]. The Contour Spacing × Relative Spacing interaction [*F*_(1, 22)_ = 9.9, *p* = 0.005] was significant, as were all of the two-way and three-way interactions between age, context, and relative spacing [Age × Context : *F*_(1, 22)_ = 16.5, *p* = 0.0005; Age × Relative Spacing: *F*_(1, 22)_ = 4.1, *p* = 0.05; Context × Relative Spacing: *F*_(1, 22)_ = 393.1, *p* < 0.0001, Age × Context × Relative Spacing: *F*_(1, 22)_ = 19, 7, *p* = 0.0002]. The significant three-way interaction implies that the effects of relative spacing and age on contour discrimination accuracy differed significantly for contours embedded among iso-oriented or randomly oriented background elements.

Next, arcsine-transformed accuracy values from the iso-oriented and random background conditions were analysed separately with 2 (Age) × 2 (Contour Spacing) × 2 (Relative Spacing) mixed-model ANOVAs. For the iso-oriented background (i.e., the upright and inverted triangles in Figure 8), the ANOVA revealed significant main effects of contour spacing [*F*_(1, 22)_ = 14.6, *p* = 0.001, η^2^_*g*_ = 0.13] and relative pacing [*F*_(1, 22)_ = 16.7, *p* = 0.004, η^2^_*g*_ = 0.07], as well as a significant Contour Spacing × Relative Spacing interaction [*F*_(1, 22)_ = 9.58, *p* = 0.005, η^2^_*g*_ = 0.04]. Accuracy was higher for contours with 3λ spacing than 6λ spacing in both groups, but this difference was slightly diminished when relative spacing was high, as accuracy for 3λ contours decreased but that for 6λ contours remained constant. The main effect of age was not significant [*F*_(1, 22)_ = 0, *p* = 0.92], nor were any of the two-way and three-way interactions with age (*F* ≤ 1.29 and *p* ≥ 0.26 in each case). Thus, contour discrimination accuracy in the iso-oriented background condition did not differ significantly between age groups.

For the random background condition (indicated by the circles and squares in Figure 8), the ANOVA revealed significant main effects of contour spacing [*F*_(1, 22)_ = 54.7, *p* < 0.001, η^2^_*g*_ = 0.25], relative spacing [*F*_(4, 88)_ = 225.5, $\hat{\epsilon }$ = 0.67, *p* < 0.001, η^2^_*g*_ = 0.67], and age [*F*_(1, 22)_ = 7.39, *p* = 0.01, η^2^_*g*_ = 0.15]. Accuracy was higher overall for 3λ compared to 6λ contours and decreased with increasing relative spacing. The Contour Spacing × Relative Spacing interaction also was significant [*F*_(4, 88)_ = 8.68, $\hat{\epsilon }$ = 0.61, *p* = 0.0001, η^2^_*g*_ = 0.05], indicating that the difference in accuracy for 3λ and 6λ spacing contours depended on the relative spacing. The significant main effect of age confirmed that older subjects showed poorer accuracy than younger subjects. As in Experiments 1 and 2, the Age × Contour Spacing interaction was not significant [*F*_(1, 22)_ = 0.13, *p* = 0.72], indicating that the effect of contour spacing did not differ for the two age groups. On the other hand, the Age × Relative Spacing interaction was significant [*F*_(4, 88)_ = 5.11, $\hat{\epsilon }$ = 0.67, *p* = 0.004, η^2^_*g*_ = 0.04]. As can be seen in Figure 8, younger and older subjects showed equally good performance at a relative spacing of 1.0. However, as distracter spacing decreased, accuracy decreased more in older subjects than younger subjects. Finally, the Age × Contour Spacing × Relative Spacing interaction was not significant [*F*_(4, 88)_ = 1.07, $\hat{\epsilon }$ = 0.61, *p* = 0.36], indicating that the increased sensitivity to relative spacing in older subjects was not significantly different for contours with 3λ and 6λ spacing.

The effect of relative spacing was analysed further by computing the linear trend scores of accuracy across relative spacing and submitting them to a 2 (Age) × 2 (Contour Spacing) mixed-model ANOVA. The grand mean differed significantly from zero [*F*_(1, 22)_ = 476.9, *p* < 0.0001], confirming the presence of a significant linear trend. The main effect of contour spacing [*F*_(1, 22)_ = 8.14, *p* = 0.009] was significant, with higher linear trend scores for 3λ spacing than 6λ spacing. The main effect of Age also was significant [*F*_(1, 22)_ = 9.92, *p* = 0.005], reflecting the increased effect of relative spacing (i.e., a greater linear trend) in older subjects. The Age × Contour Spacing interaction was not significant [*F*_(1, 22)_ = 1.74, *p* = 0.20], indicating that the age difference in the linear trend across relative spacing was similar for contours with small and large spacings.

### Discussion

This experiment examined the effect of distracter inter-element spacing on contour discrimination for contours composed of elements with small and large spacing. We found that the effect of aging depended critically on the variability of the orientations of the distractor elements. When the orientation of each distracter was selected randomly and independently, contour discrimination accuracy declined monotonically with increasing relative spacing (i.e., the ratio of contour- and distractor-element spacing), consistent with previous reports (Kovács et al., 1999; Li and Gilbert, 2002), but the effect of relative spacing differed between age groups. Specifically, reducing distracter spacing decreased older subjects' accuracy much more than that of younger subjects. For example, response accuracy was equal in both groups when contour and distracter elements had equal spacing, but accuracy was as much as 28% lower in older subjects when distractor spacing was reduced by half. On the other hand, when all of the distractor elements had the same, randomly selected orientation, older and younger subjects were equally accurate at discriminating the spiral contours, even when contour elements were spaced far apart and were interspersed with distracter elements.

As in Experiment 1, the effect of aging did not vary with contour element separation, and the increase in the effect of aging with relative spacing also did not differ as a function of contour element separation. Thus, older subjects are not disproportionately impaired at integrating contours across larger distances, even when relative spacing of contour and distracter elements is high.

Overall, our results are consistent with findings of Del Viva and Agostini (2007) who showed that younger subjects could tolerate more distracters than older subjects when detecting circular closed contours composed of a varying number of Gabors (i.e., with varying inter-element separations). They also reported a greater effect of age for contours with small spacing (3.2λ), suggesting that aging may have a greater effect on contour integration over short-range separations (Del Viva and Agostini, 2007). Similarly, if we examine performance at the largest relative spacing condition in our data—i.e., conditions with the maximum number of distracters for each contour spacing (see Figure 8, relative spacing = 2.1)—the difference in average accuracy of younger and older subjects was in fact greater for contours with 3λ spacing compared to 6λ spacing. However, this differential effect was apparent only at the largest relative spacing and was not significant when examined over the full range of relative spacings.

## General discussion

Previous research has shown that older subjects are less accurate at detecting and discriminating contours embedded in noisy or cluttered backgrounds (Del Viva and Agostini, 2007; McKendrick et al., 2010; Casco et al., 2011; Roudaia et al., 2011; Hadad, 2012). The current experiments examined how the age difference in contour discrimination accuracy varies with absolute and relative spacing between contour and distracter elements, contour element collinearity, stimulus duration, and background context. In all experiments, subjects were required to report the global orientation of a spiral contour composed of Gabor elements embedded within a homogeneous field of distracter Gabors having the same contrast, spatial frequency, and phase as the contour elements. The spiral contour was either clockwise or counter-clockwise, but its overall shape and size remained constant. The position and global orientation of the spiral varied across trials. The subjects' task in all three experiments was to report the location of the tail of the spiral. Care was taken to ensure that the alignment of the orientations of the contour elements was the only cue available for grouping the contour. A preliminary experiment indicated that young subjects required half of the contour elements to be visible in order to perform this task accurately in a condition that did not include distractor elements, which suggests that the task is a reasonable measure of how well subjects perceive the shape of an extended contour.

The current experiments revealed several novel findings. First, Experiment 1 showed that the effect of aging on contour discrimination does not vary with contour element spacing, at least over the range of spacings tested here (i.e., 2–8 times the Gabor wavelength). Second, Experiment 1 also showed that younger and older subjects are equally sensitive to disruptions in contour element collinearity at all contour element spacings tested. Third, Experiment 2 revealed that younger and older subjects can discriminate salient contours (collinear contours with small spacing) at very short stimulus durations (0.04 s). For less salient contours, older subjects showed a greater decline in performance with decreasing stimulus duration than younger subjects, consistent with previous research (Roudaia et al., 2011). Fourth, Experiment 3, revealed that the age difference in contour integration depended on the relative spacing between contour and distracter elements, rather than the absolute separation between contour elements. Lastly, both groups performed equally well when discriminating contours embedded in a dense field of iso-oriented distracters, showing that the presence of distracters *per se* is not sufficient to impair older subjects' performance.

The current study was the first to systematically examine contour discrimination for a range of contour spacings and collinearity levels in older subjects; however, the current results are consistent with several previous findings. For example, Del Viva and Agostini (2007) found that the age-related reduction in sensitivity for detecting aligned contours among distracters did not vary with contour element spacing. In addition, a recent study found that the effect of aging on contour discrimination accuracy remained constant for contours with small and large inter-element spacings (Hadad, 2012). One seemingly contradictory study found that older subjects required a greater number of contour elements to correctly discriminate the shape of a sampled contour than younger subjects, leading its authors to conclude that older adults are especially impaired at integrating contours across large separations (McKendrick et al., 2010). However, their results may also be explained by a general decline in contour discrimination with aging as observed in our study. Indeed, if older subjects' accuracy is overall lower at all contour spacings, the minimum number of contour elements required to support a criterion level of performance will also be higher than that of younger subjects. Nonetheless, examining contour integration across a range of contour spacings in Experiment 1 revealed that aging affects performance equally for a range of contour spacings, not only at larger spacings. Finally, the current results also complement findings by McKendrick et al. by showing that increasing orientation jitter impairs contour shape discrimination to the same extent in younger and older subjects.

The sensitivity to collinearity in contour integration is thought to rely on orientation-tuned neurons in primary visual cortex and long-range horizontal connections between columns of similar orientation preference (Polat, 1999; Hess et al., 2003). Neurophysiological studies in older primates have revealed significant functional changes in V1 neurons, such as increased spontaneous activity, broader orientation tuning bandwidths, and decreased signal-to-noise ratios (Schmolesky et al., 2000; Zhang et al., 2008). Such changes in human visual cortex would be expected to lead to age-related changes in tolerance to orientation jitter in contour integration tasks. However, psychophysical and electrophysiological studies in humans have found no evidence for changes in orientation tuning (Delahunt et al., 2008; Govenlock et al., 2009, 2010) or orientation discrimination ability with age (Betts et al., 2007; Delahunt et al., 2008), even for very brief stimulus durations (Roudaia et al., 2011). Thus, the finding that sensitivity to collinearity in contour integration does not change with aging is consistent with human psychophysics and electrophysiology showing preserved mechanisms for orientation encoding.

Although contour element spacing and local orientation alignment had no influence on the effect of aging on contour discrimination, older subjects were more affected by decreasing stimulus duration than younger subjects when discriminating less salient contours in Experiment 2. This change in the time needed to discriminate contours is consistent with the results of (Roudaia et al., 2011), who found that older subjects needed longer stimulus durations to discriminate contours embedded in clutter. Roudaia et al. argued that the increase in duration thresholds could not be explained by age-related reductions in retinal illuminance (Weale, 1963, 1982), because reducing stimulus luminance by 90% did not increase duration thresholds in younger subjects. Moreover, Roudaia et al. found no age difference in the amount of time needed to detect individual Gabors or discriminate their orientation. Together with the current findings, these results suggest that the longer time needed to perceive extended contours with aging results from changes in processes involved in grouping the contours and/or segregating them from the background, as opposed to processing of individual elements.

The stimulus duration required to detect or discriminate a contour among distracters varies with the characteristics of the contour and background elements, the nature of the task (detection or discrimination), the presentation of a mask, as well as subjects' previous experience with the task (Braun, 1999; Hess et al., 2001; Mandon and Kreiter, 2005; Mathes et al., 2006; May and Hess, 2007; Dakin and Baruch, 2009). On the one hand, contour detection can be very rapid, especially when stimuli are not masked (Hess et al., 2001). Mandon and Kreiter (2005) reported that monkeys can detect and discriminate contours after a masked presentation of only 0.03 s. Fast contour integration in some cases could result from the involvement of linear filters in the detection of contours comprising collinear elements with constant contrast phase (Hess et al., 2003). In the current experiments, such linear detectors may have been involved in the discrimination of collinear contours in the 2λ spacing condition, which might explain the exceptionally good performance at short durations in that condition. The absence of an age effect in that condition is consistent with our previous results showing that the contribution of linear filters for contour discrimination does not change with aging (Roudaia et al., 2011). On the other hand, when contours comprise phase-alternating elements and linear filters cannot be employed, contour detection requires stimulus durations of ≈0.1 s for straight contours and up to ≈0.5 s for curved contours (Hess et al., 2001). The earliest contour-specific neural correlate appears ≈0.15 s after stimulus onset for collinear contours and its latency is significantly delayed for less detectable contours (Mathes et al., 2006; Tanskanen et al., 2008). The current results confirmed previous findings that the processing time for contour discrimination increases with aging, especially for less detectable contours (Roudaia et al., 2011). In contrast, previous studies have found no evidence of slower processing with aging for shape discrimination (Habak et al., 2009) or detection of motion (Bennett et al., 2007). What's more, younger subjects required longer stimulus durations than older subjects to discriminate the direction of large, high-contrast drifting gratings (Betts et al., 2005, 2009). Thus, contrary to the general slowing hypothesis (Salthouse, 2000), increases in processing time are not ubiquitous in aging and the nature of age-related slowing of the dynamics of contour integration poses an interesting question for future research.

In addition to requiring longer stimulus durations to discriminate contours, older subjects showed lower accuracy when the relative spacing of contour and distracter elements was high (i.e., when contour elements were sparser than distracter elements). By varying relative spacing for contours with small and large spacing in Experiment 3, we found that the effect of aging does not depend on the absolute number or density of distracters, but rather on the relative spacing of contour and distracter elements. Previous studies have shown that contour detection is limited by the relative spacing of contour and background elements (Kovács, 1996; Braun, 1999; Kovács et al., 1999). When contour elements are spaced closer together than distracters, proximity and density cues may be used to locate and group the contour elements; when distracters are spaced closer together than contour elements, it is no longer possible to group contours based on density or proximity cues, so contour grouping must be based on the relative positions and orientations of the local elements (e.g., collinearity). Thus, contour integration becomes more difficult with increasing relative spacing because (a) greater proximity of distracters to contour elements increases the likelihood of grouping contour elements with adjacent distracters, instead of the neighboring contour elements; and (b) greater proximity of distracters to each other increases the likelihood of a chain of distractors grouping together to form false-positive contours that will compete with the target contour. Thus, differences in contour detection accuracy may be due to differences in grouping contour elements together and/or differences in the ability to select the appropriate contour among competing alternatives. Given that changes in the proximity and collinearity of contour elements had the same effect on younger and older subjects' contour discrimination performance in Experiment 1, the greater effect of relative spacing on contour integration in older subjects is likely to be caused by a reduced ability to segregate the contour from a cluttered background containing many distracters and false-positive contours.

What are the potential causes of the reduced ability to tolerate dense visual clutter in contour integration? Contour processing requires both the facilitation between responses to contour elements and suppression of responses to the background distracters (Polat, 1999; Hess et al., 2003; Dakin and Baruch, 2009; Gilad et al., 2013). The contour-related facilitation of V1 neurons is thought to be mediated by the intrinsic excitatory horizontal connections that link neurons with similar orientation preference and spatially non-overlapping receptive fields (Rockland and Lund, 1983; Gilbert and Wiesel, 1989; Amir et al., 1993; Malach et al., 1993; Stettler et al., 2002). The source of the suppression of distracters is not well known; however, Gilad et al. (2013) suggested that it may be mediated by top–down feedback on to the local inhibitory interneurons in V1, or through decreased excitatory feedback. Several investigators have suggested that age-related reductions in the efficacy of inhibitory interactions in the visual cortex (e.g., Leventhal et al., 2003) may contribute to age differences in contour integration (e.g., Roudaia et al., 2008, 2011; Casco et al., 2011). Furthermore, there is also evidence that aging may be associated with a delay in deploying top–down suppression (Gazzaley et al., 2008). To the extent that top–down suppression may be involved in contour integration, this delay may contribute to the increase in processing time needed to extract the target contour. A recent EEG study on contour detection revealed that prior knowledge that a stimulus was likely to contain a contour resulted in a decrease in posterior alpha power and fronto-posterior theta phase couplings, both of which have been proposed to reduce local inhibitory activity and increase excitability of the cortex (Volberg et al., 2013), leading to the suggestion that top–down control increases perceptual grouping by modulating the level of inhibition in early visual areas. Future studies are needed to clarify the relationship between age-related changes in levels of inhibition in visual areas, top–down control, and contour integration deficits.

Lastly, it is important to consider whether differences in cognitive strategy or perceptual decision-making may have contributed to age-related differences in performance in the current experiments. For example, in the current task, subjects may have accurately reported the location of the tail of the spiral on trials where they perceived only a part of the spiral by correctly extrapolating the rest of the spiral's shape. It can be argued that the small main effect of age observed in Experiment 1 may be due to differences in the use of such a cognitive strategy. However, such differences can not easily account for the increase in the effect of aging at short durations in Experiment 2, or the greater effect of relative spacing in older subjects in Experiment 3. Perceptual decision-making refers to the process of acquiring information from sensory neurons to make an appropriate response (Gold and Shadlen, 2007). Previous studies analysing response times in several perceptual tasks have found that older subjects were as efficient as younger subjects at accumulating sensory evidence in the decisional process, and that their slower performance was mainly due to more conservative response criteria and non-decisional processes (Ratcliff et al., 2006; Kühn et al., 2011). If the quality of contour-related sensory evidence was the same in both groups, but the efficiency of gathering the evidence was poorer in older subjects, decreases in stimulus duration should have had an equal detrimental effect on older subjects across all contour types, which was not the case. Moreover, given that subjects were not encouraged to respond quickly in the current experiments, age-differences in response criteria should not have affected performance in the current experiments. In sum, although differences in the use of cognitive strategies or perceptual decision making may contribute to age-differences in contour discrimination, they are not sufficient to explain the full pattern of results. Instead, the current findings are more consistent with age-related changes at the level where local elements are grouped into perceptual wholes.

## Conclusion

The current study replicated previous findings of impaired contour integration with aging and revealed that the effect of aging does not vary with contour element spacing or with the local orientation alignment of contour elements. Instead, the effect of aging on contour integration increased with increasing contour-distracter relative spacing. Thus, perceptual grouping of contours is especially vulnerable to distracters with aging.

### Conflict of interest statement

The authors declare that the research was conducted in the absence of any commercial or financial relationships that could be construed as a potential conflict of interest.
